# Examining intentions to engage in sun protective behaviors among Latino day laborers: An application of the theory of planned behavior

**DOI:** 10.34172/hpp.2021.45

**Published:** 2021-08-18

**Authors:** Javier F. Boyas, Jana L. Woodiwiss, Vinayak K. Nahar

**Affiliations:** ^1^School of Social Work, University of Georgia, 279 Williams St., Athens, GA, 30602, USA; ^2^Department of Dermatology, University of Mississippi Medical Center, 2500 N., State St., Jackson, MS 39216, USA

**Keywords:** Skin neoplasms, Intention, Sun screening agents, Health behavior, Hispanic Americans, Primary prevention

## Abstract

**Background:** The past two decades has revealed an unprecedented increasing incidence of skin cancer within the Latinx population. Although Latino day laborers (LDLs) are at heightened risk for developing skin cancer because of the outdoor work in which they engage, there is limited research examining their intentions to engage in sun protective behaviors (SPBs). Therefore, this study sought to assess the explanatory power of the theory of planned behavior (TPB) to identify attitudinal, subjective norms, and perceived behavioral control factors associated with intentions to engage in SPB among LDLs.

** Methods:** This cross-sectional retrospective study consists of a non-random convenience,community-based, sample of 137 LDLs residing in Mississippi and Illinois. Data were collected using a self-report survey centered on health practices and sun-protective behaviors.

**Results:** Findings revealed that five significant factors shaped intentions to engage in SPBs, including barriers to engaging in SPBs (β =.30, P<0.001), benefits of engaging in SPBs (β =.27,P<0.001), education (β=0.20, P<0.01), and acculturation (β=0.18, P≤0.05). The independent variables tested in the model accounted for 42% of the change in intentions to engage in SPBs.

**Conclusion:** This study demonstrates TPB’s usefulness for predicting future intentions to engage in SPBs among LDLs. Moreover, the strongest factor associated with predicting intentions to engage in SPBs among LDLs was perceived behavioral control. Thus, since SPBs are malleable, emphasis is placed on implementing interventions for this population that promote intentions and address perceived behavioral control.

## Introduction

Skin cancer remains the most pervasive cancer threat in the United States (U.S.).^1^ Basal cell carcinoma (BCC), a frequently diagnosed type of skin cancer, is estimated to account for 4.3 million diagnosed cases in the U.S. each year.^2,3^ BCC is also a regular form of skin cancer among the Latinx population.^4^ Despite being at lower risk for acquiring skin cancer, incidence rates of skin cancer among the Latinx population have risen by as much as 20% in the past two decades.^3,5,6^ Moreover, data from the Centers for Disease Control and Prevention suggest that in 2017, 70% of all new skin cancer cases among U.S. racial and ethnic minorities occurred within the Latinx population.^7^ These statistics are concerning because, while non-Latinx Whites have a higher likelihood of being diagnosed with skin cancer, a higher proportion of Latinx people diagnosed with skin cancer are more likely to die from it.^3^

It is well established that elevated sun exposure is a significant risk factor for the genesis of non-melanoma skin cancer.^8^ Since so much time is spent outside, outdoor workers, such as Latino day laborers (LDLs), are at heightened risk of developing non-melanoma skin cancer compared to indoor workers or the broader general public.^9-11^ The amount of sun exposure is a major concern because most jobs that LDLs occupy require working outdoors for extended periods of time.^12^ On average, outdoor work is estimated to account for 75% of a workday,^9^ which partially explains why outdoor workers are exposed to higher than recommended doses, or excess of the limit, of sun exposure.^13^ Despite the elevated risks of working outdoors, existing research suggests that Latino outdoor workers do not adequately protect themselves. For example, a national survey of Latino outdoor workers revealed that 69% of participants never used sunscreen or rarely used sunscreen while at work.^14^ A key limitation associated with that study was that it only assessed sunscreen use, failing to account for broader uses of sun protective behavior (SPB) among this subpopulation. Thus, it is maintained that continuous exposure to UVR is one of the most often overlooked occupational risks associated with day labor, but it also one of the more dangerous threats to the health and well-being of LDLs.

While additional research is needed to establish use of broader SPBs among LDLs, more can be learned by understanding their intentions to engage in SPBs since intent to engage in any action is the prominent determinant of the behavior, particularly SPBs.^15,16^ One of the more popular theories used to better understand health behaviors is the theory of planned behavior (TPB), which assumes that the higher the value a person places on the positive outcome associated with performing SPBs, the greater the likelihood of a behavior being carried out.^17,18^ Behavioral intention is an assessment of an individuals’ motivation to carry out the desired behavior.^17^ Behavioral intentions are the central tenet of TPB, which is influenced by three constructs associated with TPB: attitudes, subjective norms, and perceived behavioral control. Attitude relates to the individual’s perception of a behavior; it assesses the positive or negative outcome of performing SPBs.^17^ In turn, attitude is guided by behavioral beliefs and evaluation of possible outcomes. Subjective norms are the individual’s perceptions of shared pressures to perform SPBs.^17^ Subjective norms are determined by how LDLs believe those around them, such as supervisors and coworkers, feel about SPBs. Thus, LDLs are more likely to conform to what others in the workplace expect from them. Perceived behavioral control represents the degree to which LDLs may find it easy or less difficult to carry out SPBs.^17^ The broader literature suggests that attitudes,^19-22^ social norms,^20,23,24^ and perceived control^22,25-28^ factors are significantly associated with intentions to engage in SPBs.

To date, few studies have explored the sun protection strategies of LDLs, despite the amount of time they spend outdoors in a typical workday. The following underscores the uniqueness of the present study. First, to the authors’ best knowledge, there has not been a study carried out that has examined the SPB intentions of LDLs. This is a significant knowledge gap because behavioral intentions are a major driving force for engaging in SPBs. Second, this was the lone study to test whether the TPB has the explanatory power to predict intentions to engage in SPB among LDLs. It is well established that this theory has predictive power related to SPBs across multiple populations; however, this knowledge is not available as it relates to LDLs. This was the first study to examine whether workplace norms, perceived barriers to engaging in SPBs, individual health values, and self-efficacy were salient in predicting intentions to engage in SPBs among LDLs. This is an essential consideration because the question of applicability of theory across populations, settings, contexts, and behaviors is required if a theory is to build generalizability, and replication addressing the limits of a theory should not be of lesser importance than a new discovery.^29^ Last, it is well established that developing skin cancer is malleable, and primary prevention strategies can help mitigate the risk. Thus, research that is specific to a population or context is essential. Using the TPB framework, this study sought to explore what attitudinal, subjective norms, and perceived behavior control factors promoted or impeded the intentions of using SPBs among LDLs in Mississippi and Illinois. This study also controlled for salient sociodemographic characteristics such as age, education level, socioeconomic status, skin type, and acculturation.^24-26,30-38^

## Materials and Methods

### Study design and participants 

A cross-sectional, retrospective survey research design was used in this study. It included a non-random, convenience community-based sample. Participants were LDLs residing in the U.S., in either the state of Mississippi or Illinois. In Mississippi, the data were collected in four different cities: Hernando, Holly Springs, Oxford, and Southaven, whereas in Illinois, the data were collected only in Chicago. Diverse recruitment strategies were used in both states because LDLs are a hard-to-reach population. Network-based referrals were sought from collaborating social service and healthcare providers serving the local Latino population throughout Northern Mississippi. Active recruitment strategies were used such as venue-based sampling, snowball sampling, and social networks. Venue-based face-to-face sampling took place at multiple construction sites. During face-to-face recruitment, all study participants were asked if they knew someone else who could be a potential participant for this study. This strategy was needed because the size of the Latino population in these cities is not large, and day laborers cannot congregate in one location the way it is done in larger metropolitan areas. Passive recruitment efforts included reaching out to participants via advertisements (printed in English and Spanish) and posting flyers in places where day laborers often visit (e.g., Mexican restaurants, cleaners, churches, and construction sites). In Illinois, the data collection was limited to the northwest side of Chicago. All of the participants were recruited face-to-face from three popular, informal street corners where LDLs are frequently known to gather in search of employment.

For LDLs to be eligible to participate in the study, they had to: (1) self-identify as either Hispanic or Latino, (2) be at least 18 years or older, (3) actively seek informal and contingent employment, (4) have no cognitive limitations, (5) live in a community dwelling, and (6) live in Mississippi or Illinois. A total of 137 participants were included in the analysis, with the majority residing from Mississippi (*n* = 77). The sample size is consistent with other studies focused on LDLs.^39-41^ To determine the power in the present study’s analysis, or type II error rate, a *post hoc* achieved power analysis was conducted using G*Power.^42^ The main outcome used in the multiple regression model was intentions to engage in SPBs. An *F* test for multiple linear regression, fixed model *R2,*deviation from zero was computed. In terms of *f*2, the effect size was the adjusted squared variance from the regression model results, which was 0.42. Adding this number to G*Power, it recalculated the f2 and changed the effect size to 0.724. The alpha was set at 0.05, the study’s sample size was 137, and predictors included in the model set at 10. Results indicate that a 1.00 power was achieved in the present analysis, which suggests that the sample size was acceptable to reach a high level of power and lessen the probability of making a type II error.

Data were collected in both states in 2014 between July and November. During this period, the research team intentionally collected data on sun-safe practices to limit the possibility of participants’ recall bias. For example, due to the continued number of sunny days, data collection in Mississippi extended well into November. Conversely, in Illinois, data collection was discontinued in October because day laborers were no longer exposed to prolonged periods of sunshine. The administration of the survey was led by the lead author and three research assistants (RAs). To establish data collection consistency, the three RAs participated in a two-hour training on proper data collection practices and interviewing techniques. The research team was made up of bilingual (English-Spanish)/bicultural individuals. Participants received a research honorarium of $20.00. All participants completed informed consent documentation to participate in this study. The University of Mississippi’s Institutional Review Board approved the procedures of this study consistent with the Helsinki declaration.

### Measures

All participants completed a self-report questionnaire, which was available in Spanish or English, depending on the respondent’s language preference. Questionnaires were completed in a little under 60 minutes. Respondents were offered the option of hearing an oral reading of the survey. This was done to dissuade participants with limited literacy from not participating. Only a few respondents opted to take up this option. Validated scales were mainly used to establish the self-report questionnaire used in this study.^36,43-49^ Several of these instruments were available in Spanish.^43,45,47^ When they were not available, the research team translated them. As part of the translation, the research team was intentional about using words in the questionnaire that most people could read and understand, including those who did not possess high literacy levels. According to the Flesch-Kincaid Readability rating, the translation process produced a survey that called for a 5^th^ to 6^th^ grade reading ability.^50^

### Dependent variable

A five-item scale was used to measure the behavior intention to engage in SPBs while at work. Each item used a five-point Liker-type scale (1 = “strongly disagree”; 5 = “strongly agree”).^49^ The five items were totaled to create an index where higher scores represented increased intentions to engage in SPBs. The intention to engage in SPBs measure achieved an acceptable level of internal reliability in this study (Cronbach’s α = 0.78).

### Independent variables

There was a total of six sociodemographic variables measured for this study, including age, education, annual income, immigration status in the U.S., number of years living in the U.S., perceived skin type. Age and number of years living in the U.S. were both single continuous sociodemographic measures. Education was measured in two ways, first as a dichotomous measure (0 = completed less than high school and 1 = completed high school and beyond), and in terms of where the respondent’s education took place. The respondents were asked two questions to identify the location of education. First, if they were born outside of the U.S, and if they were educated in their country of origin (0 = no, educated in the U.S. and 1 = yes, educated in my country of origin). Yearly income and perceived skin type were both single ordinal measures. In the analysis, yearly income was dichotomized due to limited variance from respondents (0 = earned less than $ 20 000 and 1 = earned more than $20 000). Perceived skin type was also dichotomized (0 = seldom burn, rarely burns, easily tans to fairly brown pigment, tans very easily, and not ever burn, considerable skin pigmentation and 1 = persistently burn, does not tan, frequently burn, struggle to tan, and occasionally moderate burn, increasingly bronzes to a pale brown).

The Short Acculturation Scale for Hispanics (SASH) was used to assess acculturation levels.^45^ This scale included a total of four questions that used a 5-point Likert-type scale: 1 = (only Spanish) to 5 = (only English). For example, one question invited respondents to state which language they frequently spoke at home. The four items were added together to create an index that identified the level of acculturation for participants. Respondents with higher scores were more acculturated. The SASH achieved a high level of internal reliability (Cronbach’s α = 0.91) in this study.

To measure perceived benefits of SPBs, participants were asked to appraise their level of concurrence with three statements.^44,46^ For example, one statement focused on how the participant agreed that a benefit of protecting oneself from the sun was to decrease aging of the skin. Level of agreement was rated on a Likert scale (1 = “strongly agree”; 5 = “strongly disagree”). A Cronbach’s α = 0.76 was achieved by the perceived benefits measure in this study.

A total of 12 statements were used to measure perceived barriers to SPBs. For example, one statement suggests that “Use of sun protection measure is time consuming.” The 12 statements were rated using a Likert-rated scale (1 = “strongly disagree”; 5 = “strongly agree”). Higher scores signifying greater perceived barriers to SPBs. In this study, an acceptable Cronbach’s α = 0.72 was achieved by the perceived barriers measure.

Seven items were utilized to measure self-efficacy.^44,46^ For example, one item gauged the extent to which someone was confident in being able to seek shade while working outside for more than 15 minutes between 10 AM and 4 PM. Response options went from 1 to 10 (1 indicating “not at all confident” and 10 indicating (“highly confident and certainly can do”). The seven items were totaled, with higher scores representing higher efficacy levels. The Cronbach α = 0.88 achieved a high level of reliability.

To determine how much value LDLs placed on health, the four-item health value scale was used.^51^ Examples of items within the health value scale include: “if you don’t have your health, you don’t have anything” and “there are few things more important than good health.” These statements were rated using a Likert-rated scale (1 = “strongly agree”; 7 = “strongly agree”). After aggregating these four items, high scores suggest a higher value was placed on health. The health value scaled achieve a high Cronbach’s α = 0.89.

To gauge a respondent’s knowledge about risk factors associated with skin cancer, a seven-item scale was used.^43^ For example, one statement was whether having dark-colored skin was a skin cancer risk factor. Three response option were available: (0 = “no,” 1 = “yes,” 2 = “don’t know”). Respondents received a “1” when they answered the question correctly. The seven items were aggregated to produce an index, where higher scores implied the respondent possessed more knowledge about skin cancer risk factors. Results of the Cronbach alpha suggest moderate internal consistency was achieved (α = 0.80).

A single, continuous measure was used to measure time spent outdoors. Respondents were asked to write down the number of hours they spent outdoors at work during peak sun times.

The authors created the two workplace support items.^36^ The items asked participants to ascertain how much they believe their (1) supervisor(s) and their (2) coworkers participate in SPBs. Each item was measured on a Likert-type scale (1 = never, 5 = always).

### Data analysis strategy

The statistical analyses in this study were calculated using IBM SPSS version 22.0 (IBM Corp. Armonk, NY, USA).^52^ Statistical significance was set at.05. Overall, less than one percent of the data were missing; thus, the pairwise deletion method was used to treat missing data. Descriptive statistics were utilized to establish key demographic characteristics associated with this study’s sample. Cronbach’s alpha was estimated for measures presumed to infer an implicit concept. A zero-order correlation was computed to determine the directionality and strength of the relationship between intentions to engage in SPBs and all independent variables. To examine the relationship between intentions to engage in SPBs and independent variables, a multiple linear regression model was calculated. Regression diagnostics were examined and none of the Gauss-Markov ordinary least squares assumptions were violated.^53^ To build the multivariate model, only predictors that were statistically significant (*P* ≤ 0.05) at the bivariate level were included as predictors. In the multivariate model, all variables were entered simultaneously. To present the results of the multivariate model, the independent variables were shown by the strength of influence on intentions to engage in SPBs.

## Results

A total of 138 LDLs took part in the present investigation, but one participant was deleted from the analysis because she was a female. The majority of the respondents were of Mexican origin (*n* = 97), followed by respondents from Central America (*n* = 24). LDL averaged 35 years of age. The study’s sample reported mostly being of undocumented legal status (*n* = 50) and foreign-born participants reported being in the country for a little over a decade. Almost half of the LDLs reported achieving less than an eighth-grade education and many foreign-born LDLs reported being educated in their country of origin (*n* = 115). In terms of marital status, the two largest groups were LDLs who were married (*n* = 69), followed by those who were single (*n* = 45). Yearly income was fairly low among LDLs, many earned less than $20,000 annually (*n* = 95). Most LDLs worked in outdoor construction, (*n* = 57), followed by landscaping (*n* = 25). LDLs reported spending close to five hours outside while at work during peak sun hours. The overwhelming number of LDLs reported never receiving a skin exam (*n* = 127). Among LDLs, the clearest intention to engage in SPB while working was to find shade during the peak sun hours, while the slightest intentions reported was to apply sunscreen. In terms of advantages of safeguarding against the sun during peak hours while at work, LDLs reported the highest endorsement for protecting themselves from the sun to lessen the possibility of getting skin cancer. LDLs identified that the greatest barrier to protecting themselves from the sun was that the process was not always convenient.

### Bivariate results

Results of the zero order correlations indicate that intentions to engage in SPBs was significantly linked to self-efficacy, health beliefs, barriers to engaging in SPBs, benefits of using SPBs, acculturation, legal status, education, being educated in their country of origin, skin tone, and workplace support for SPBs. LDLs who perceived greater benefits to employing SPBs (*r* = 0.38, *P* ≤ 0.001) also reported higher intentions to engage in SPBs. However, LDLs who noted more barriers to using sun safe strategies (*r* = -0.43, *P* ≤ 0.001) also conveyed decreased intentions to engage in SPBs. LDLs who endorsed higher intentions to engage in SPBs were more acculturated (*r* = 0.40, *P* ≤ 0.001); had higher levels of self-efficacy in being able to use SPBs (*r* = .32, *p* = <.001); were more educated (*r* = 0.27, *P* ≤ 0.05); were of legal status (*r* = 0.26, *P* ≤ 0.01); professed stronger health beliefs (*r* = 0.21, *P* ≤ 0.05) and were at greater risk because of skin reaction to sun (*r* = 0.18, *P* ≤ 0.05). LDLs who stated they were educated in their home country also reported lower intentions to engage in SPBs (*r* = -0.30, *P* ≤ 0.001). LDLs reported greater intentions to engage in SPBs when they thought their supervisors (*r* = 0.21, *P* ≤ 0.05) and coworkers (*r* = 0.34, *P* ≤ 0.001) also engaged in SPBs at work. Age, income, years of length in the U.S., and knowledge of cancer risk factors were not significantly correlated with intentions to engage in SPBs. Thus, these independent variables were excluded from further analyses.

### Multivariate results

Multiple linear regression with intentions to engage in SPBs as the outcome yielded a significant model of five significant factors: *barriers to engaging in SPBs* (*β* = -30, *P* < 0.001), *benefits of engaging in SPBs* (*β* = 27, *P* < 0.001), *perceptions of how much their coworkers engage in SPBs* (*β* = 0.22, *P* < 0.01), *education* (*β* = 0.20, *P*< 0.01), and *acculturation* (*β* = 0.18, *P* ≤ 0.05), (see Table 1). The regression model accounted for 46% of the change in intentions to engage in SPBs (adjusted *R*^2^ = 0.42). LDLs reporting greater barriers to engaging in SPBs was expected to decrease intentions to engage in SPBs by 0.30 units. Additionally, reporting more benefits of engaging in SPBs was associated with a 0.28 increase in greater intentions to engage in SPBs. LDLs who believed their coworkers engaged in SPBs increased their intentions to engage in SPBs by 0.22 units. Results show that having achieved at least a high school diploma was associated with a 0.20 unit increase in greater intentions to engage in SPBs. LDLs who reported being more acculturated was associated with a 0.18 increase in greater intentions to engage in SPBs. In the multivariate model, confidence in engaging in SPBs, perception of how much their supervisors engage in SPBs, legal status, health beliefs, being educated in one’s country of origin, and being at greater risk because of skin reaction to sun lost statistical significance.

**Table 1:** Multivariate regression model summary of predictors of intention to engage in SPBs among LDLs (*N* = 137)

**Variables**	***B ***	**95% CI**	***β***	***P *** **value**
Barriers to use SPBs	0.122	0.065-0.179	0.303	0.001
Benefits of SPBs	0.458	0.691-0.911	0.267	0.001
Support of SPBs by co-workers	0.946	0.244-1.648	0.220	0.009
Education	2.063	0.699-3.426	0.203	0.003
Acculturation	0.201	0.016-0.386	0.179	0.033
Self-efficacy	0.043	-0.007-0.094	0.120	0.093
Support of SPBs by supervisors	-0.505	-1.302-0.291	-0.102	0.212
Health beliefs	0.062	-0.126-0.250	0.050	0.517
Age	-0.013	-0.083-0.057	-0.026	0.705
Legal status	0.768	-0.779-2.316	0.076	0.327
Perceptions of being at greater risk because of skin reaction to sun	0.061	-1.393-1.515	0.006	0.934

F(11) = 9.850, *P* ≤ 0.001, R2 (Adjusted R2) =.468 (0.421) Outcome variable is intentions to engage in SPBs; B = unstandardized coefficient; CI = confidence interval; β = standardized coefficient; *P* = level of significance.

## Discussion

One of the most effective means of reducing the genesis of skin cancer is engaging or intending to engage in SPBs. Therefore, this research sought to explore the utility of TPB in predicting intentions to engage in SPBs among LDLs in Mississippi and Illinois. To date, no study has examined the intentions to engage in SPBs among LDLs, nor has this outcome been explored using a TPB lens. This is a major gap in the literature, given that a considerable number of LDLs spend their workday working outdoors during peak sun hours. As a result, they become exposed to intense and continuous sun exposure, which may ultimately advance the occurrence of skin cancer. Thus, continuous sun exposure remains one of the most overlooked occupational hazards associated with day labor.

Results provide support for using TPB as a theoretical lens to understand intentions to engage in SPBs among LDLs. However, each construct of TPB had a varying impact on the intentions to engage in SPBs. The findings indicate that intentions to engage in SPBs are associated with attitudes, normative beliefs, and perceived control. However, the strongest factor associated with predicting intentions to engage in SPBs among LDLs was perceived behavioral control. Consistent with other studies,^29,54^ perceived barriers to engaging in SPBs were significantly associated with lower levels of intentions to engage in SPBs. Within a situation of restrictions to carry out a behavior, such as in the workplace, LDLs with higher perceived control levels and higher intentions would be more likely to achieve SPBs than LDLs with lower perceived control levels.^18^ It is the perception of the presence or absence of barriers that shaped the LDLs ability to perform SPBs.

Data suggest the most frequently cited barriers among LDLs were: it was inconvenient to safeguard against the sun, often unable to remember to safeguard against sun exposure, and not always being able to apply sunscreen again. These barriers might be challenging to alter because LDLs are not in complete control of engaging in SPBs. A qualitative study of outdoor workers revealed that respondents reported not having the latitude to rearrange their tasks to better protect themselves from working outdoors during the peak sun hours, mainly because this is an implicit task of working in an outdoor profession.^11^ Moreover, given the informal workplace structure of day labor, engaging in SPBs may not be convenient or possible because their time at work is regulated by someone else. Researchers have established the exploitive conditions in which LDLs work, such as working forced long hours, experiencing immense pressure to complete their tasks quickly due to productivity-based pay, and being denied opportunities during the work day to eat something and consume a drink.^55-57^ It is plausible that LDLs are not given much time to apply, or reapply, sunscreen, take shade during peak sun hours, or be given a moment to think about what strategy(ies) they could use to protect themselves from sun exposure throughout the day.

Consistent with existing studies,^20-22,29^ more positive attitudes or higher perceptions of engaging in SPBs as beneficial was also significantly associated with increased intentions to engage in SPBs. However, most of the LDLs recognized that a benefit of engaging in SPBs was protecting themselves to lower the threat of developing skin cancer. This result contrasts what has been found in other research that involves the broader Latinx population, suggesting that this population marginally agrees with statements regarding sun protection benefits.^34^ These mixed results should be explored in future research. Researchers ought to continue exploring the fidelity of the assessments used in research to identify this population’s true beliefs and attitudes about the benefits of SPBs. More nuanced measures are needed that determine the accurate meaning and significance the Latinx population places on SPBs.^58^

Findings also suggest that intentions to engage in SPBs are significantly associated with perceived workplace subjective norms. Consistent with the broader literature,^11,59,60^ increased perceptions that coworkers engage in SPBs are associated with increased intentions to engage in SPBs. At the bivariate, increased supervisory support was also significantly associated with intentions, but this factor lost significance when other factors were accounted for in the multivariate model. It appears that LDLs’ intentions to engage in these behaviors are associated with how much their peers engage in SPBs.

The findings also show that acculturation shared a significant association with intentions to engage in SPBs. These results suggest that intentions to engage in SPBs were higher among LDLs who were more acculturated. However, when parceling the items, acculturation showed mixed effects on multiple items included in the intentions to engage index. The results are consistent with existing research in that less acculturated LDLs have a lower likelihood of using sunscreen, which is more of a U.S. cultural norm.^27,61^ However, other research shows that more acculturated individuals are more inclined to seek shade, wear sun-protective clothing when outdoors, have greater perceived suntan benefits, and have lowered perceptions of the threat of getting skin cancer.^27,38^ It may be that acculturation has positive and negative effects on intentions to engage in SPBs. Thus, the correlation involving acculturation and intentions to engage in SPBs is still not well-defined. Researchers should continue to examine this complex relationship longitudinally and qualitatively to determine the trajectory of engaging in SPBs and acculturation over time.

Although this study was the first to explore how the TPB shapes intentions to engage in SPBs among LDLS, several limitations should be acknowledged. First, causal inferences cannot be deduced because of the cross-sectional nature of the research design. To establish more causal evidence between TPB components and SPBs, future studies should utilize a more robust research design. Second, data were collected from only two states using a nonrandom, convenience sampling method, thereby limiting this study’s generalizability. For future research, randomized sampling with a larger sample of LDLs is recommended to better represent this subpopulation more accurately. Another limitation is recall bias, which may been present because of the retrospective nature of the measures. Fourth, due to the small sample of participants from some Latinx subgroups, the data were also analyzed in aggregate. Not stratifying the analysis by subgroups may overlook some of the within-group differences that exist within the polylithic Latinx community. Fifth, the acculturation measure assessed mainly linguistic acculturation, which only encompasses one aspect of this multidimensional construct. Future studies replicating this study must also include other aspects of acculturation.

### Implications

Several important implications can be drawn from this study. First, LDLs reported low future intentions to use sunscreen as an SPB. This finding is problematic because sunscreen compliance can reduce solar damage and decrease the risk of contracting skin cancer.^62^ Moreover, for LDLs who did use sunscreen, they reported it was harder to reapply. Thus, this group, and most likely other workers working alongside them, need continuous reminders to use and reapply sunscreen throughout the workday. Using cues-to-action strategies may address this issue by facilitating improved engagement in SPBs, such as posting flyers around the worksites and perhaps using short message service (SMS) to send text reminders throughout the day, particularly during peak sun times. Evidence suggests that electronic interventions, such as mobile phones, can promote skin cancer prevention behaviors.^63,64^ Additionally, the messages to engage in SPBs can come from medical personnel. In earlier studies, intentions increased through physician recommendation.^21^

Second, perceived barriers had the strongest effect on reduced intentions to engage in SPB. Overcoming these barriers will be challenging because the spaces in which LDLs work often lack health and safety protections and are places where workers must tolerate substandard work conditions.^65,66^ Results from this study serve as a call to action for the U.S. Office of Safety and Health Administration (OSHA) to provide closer surveillance and regulate the worksites that employ LDLs. Additionally, OSHA does take a position on addressing SPBs; however, much of their emphasis put the responsibility of protecting oneself purely on the workers. Much of their printed literature urges individuals to take up SPBs. However, that task cannot be strictly left up to workers because they do not have the liberty of controlling their time. OSHA should train and expect organizations to contribute to this effort because they can manage their workers’ time. Third, results suggest that LDLs are more likely to increase their intentions to engage when they perceive a work safety cultural norm where coworkers and supervisors engage in SPBs. While the LDLs are part of the informal labor market, their employers should create a work culture that values their workforce’s health and safety by supporting and spearheading sun-protective strategies. Doing so would help not only LDLs, but also full-time outdoor workers. Promoting SPBs in the workplace will help protect all outdoor workers, particularly those workers at elevated risk of excess solar exposure. Fourth, work centers, such as the National Day Labor Organizing Network, should embrace the notion that excess solar exposure is an occupational health hazard faced by LDLs. Work centers have dealt with some of the structural changes needed to improve the work-related security and wellness of LDLs.^12,65,67^ However, they have not advocated for making changes in the workplace that address SPBs. Last, the findings suggest that sun protection interventions targeting LDLs can use TPB as a possible framework. Since SPBs are malleable, emphasis is placed on interventions that increase intentions and address perceived behavioral control. The literature shows that interventions that increase an individual’s locus of control will be more successful in engendering behavioral modification if the intention prompts intentions or further elevates them.^23,68^

Although several implications were drawn from the findings, future research should explore the following. First, qualitative studies should be carried out exploring in-depth how the context of the workplace milieu affects autonomy related to engaging in SPBs. Specifically, what distal and proximal barriers exist in the workplace? Second, more research is needed to ask LDLs specifically what would motivate them to change their intentions and actual skin protection behaviors. Finally, given that workplace supports enhanced intentions to engage in SPBs, what workplace strategies can be developed that can be carried out by supervisors and fellow workers to promote primary prevention SPB strategies among LDLs? For example, would training supervisors and fellow workers as peer health promoters help LDLs adopt sun-safe behaviors?

## Conclusion

The present study shows the TPB’s usefulness for predicting future intentions to engage in SPBs among LDLs and underscores the predictive significance of perceived behavioral control, subjective norms, and attitudes. However, that is not true for self-efficacy, which did not significantly predict intentions to engage in SPBs. Results suggest that promoting LDLs’ perceptions of behavioral control may be the most robust means of modifying their SPBs. However, these results may be context specific. In the case of LDLs, it may not matter if they express confidence in engaging in SPBs. Data suggest a number of these men expressed perceptions of losing locus of control. For LDLs who face work exploitation and have no latitude in the workplace, the perceived lack of control trumps confidence when it comes to intentions to engage in SPBs. Given the volatile and unregulated work environment in which LDLs work, health promotion professionals will find it challenging to help them overcome perceived control barriers because of the non-compliant work standards that seem to permeate within the informal labor market. Until work sites receive ongoing monitoring and enforcement of labor standards, LDLs will continue to face inequities in the workplace that give way to unequal health consequences, which in this case means continuing higher mortality rates among this population when contracting skin cancer.

## Acknowledgments

The authors would like to acknowledge the day laborers who contributed to this study. Their participation was invaluable.

## Funding

No external funding was secured for this study.

## Competing interests

The authors confirm no conflicts of interest.

## Ethical approval

Procedures carried out in studies involving human participants were in accord with the ethical tenets outlined in the 1964 Helsinki declaration and its later amendments. Informed consent was obtained from all participants in this study. Every protocol associated with the present study was authorized by the Institutional Review Board at the University of Mississippi.

## Authors’ contributions

JFB developed the concept and design of this research project; helped translate survey instrument, participated in data collection and analysis, and participated in the manuscript editing and review process. JLW participated in the literature search, manuscript preparation and manuscript editing and review process. VKN participated in the concept and design, data collection, and manuscript preparation. All three authors have examined, confirmed, and agreed to the submission, and they are responsible every aspect of its accuracy and honesty in accordance with ICMJE criteria.
